# Spinach SoNRT3 Interacts with SoNRT2a to Improve Low-Nitrogen Tolerance via Nitrate Uptake and Root Growth

**DOI:** 10.3390/plants14142126

**Published:** 2025-07-10

**Authors:** Zihang Chen, Xitong Qu, Minhua Zhao, Jiapeng Shui, Xinyue Liu, Xiaofeng Cai, Chenxi Xu, Xiaoli Wang

**Affiliations:** Shanghai Collaborative Innovation Center of Plant Germplasm Resources Development, College of Life Sciences, Shanghai Normal University, Shanghai 200234, China; gamuterniff@outlook.com (Z.C.); qxt19991114@outlook.com (X.Q.); asd199608163@163.com (M.Z.); s13637056678@163.com (J.S.); 18328932009@163.com (X.L.); cxf0012@163.com (X.C.); chenxixu@shnu.edu.cn (C.X.)

**Keywords:** spinach, nitrate transport, NAR2, NRT3, NRT2, nitrogen use

## Abstract

High nitrogen use efficiency is crucial for enhancing spinach’s tolerance to low nitrogen stress and minimizing nitrate accumulation. Here, we report that SoNRT3, a NAR2 family protein, modulates nitrate uptake and plant growth under low-nitrate conditions. SoNRT3 expression was induced by low nitrate availability in roots and prolonged nitrogen deficiency in shoots. Compared to wild-type *Arabidopsis thaliana*, lines overexpressing *SoNRT3* exhibited higher root fresh weight, activities of nitrogen reduction/assimilation-related enzymes, tap root length, and total root diameter under low-nitrate (0.25 mM) conditions. *SoNRT3* silencing reduced taproot length, lateral root number, shoot/root biomass, and ^15^${NO}_{3}^{-}$ uptake in spinach grown under low-nitrate conditions. *SoNRT3* partially compensated for ^15^${NO}_{3}^{-}$ uptake in *atnrt2.1* and *atnrt3.1* mutants. Transcriptome analysis showed that *SoNRT3* may enhance nitrate uptake and root development by promoting the expression of high-affinity nitrate transporters, nitrogen assimilation, auxin signaling, and cell differentiation. Additionally, SoNRT3 can interact with a spinach NRT2 family protein (SoNRT2a), whose transcription level was also induced by low N and N deficiency. Together, this study clarifies the key roles and regulatory network of SoNRT3 in low-nitrate tolerance, which contributes to a novel understanding of nitrate utilization in spinach.

## 1. Introduction

Spinach (*Spinacia oleracea* L.) is an important leafy vegetable cultivated all over the world. Nitrate nitrogen serves as an essential element for the growth and development of spinach. An adequate nitrate supply helps spinach yield increase, but high levels of accumulated nitrate may reduce spinach nutritional quality and pose risks to human health and environmental pollution [1,2]. This challenge is particularly acute in controlled-environment agriculture systems (e.g., vertical farms), where balancing productivity with food safety demands optimized nitrate use efficiency [3]. Given that lighting is a major cost factor in controlled-environment agriculture systems, directly influencing nitrate content (inversely proportional to photosynthetic photon flux density), developing spinach varieties with improved nitrogen utilization efficiency could significantly reduce dependency on intensive lighting while minimizing nitrate accumulation [4,5]. Therefore, enhancing nitrogen use efficiency (NUE) under limited nitrate availability may represent a dual-purpose strategy for maintaining yield while minimizing nitrate accumulation. It is well known that most plant-acquired nitrates are actively taken up and transported via nitrate transporters (NRTs). Therefore, optimizing the activity of nitrate transporters is a prerequisite for plants to improve nitrogen utilization [6].

Plants actively uptake and transport nitrate depending on a series of nitrate transporter (NRT) members, including nitrate transporter 1/peptide transporter (NRT1/PTR, also known as NPF [7]), NRT2, and NRT3 (also known as NAR2) [8]. Among them, some NRT2 members are generally responsible for the high-affinity transporter system (HATS, nitrate concentrations < 1 mM) based on their different working concentrations of nitrate [9]. NRT2-mediated HATS usually requires the assistance of NRT3 [10]. The chaperone function of NRT3 (also known as NAR2) in high-affinity nitrate transport was first identified in the single-celled green algae *Chlamydomonas reinhardtii*. In this alga, CrNRT2.1 and CrNRT2.2 lack autonomous high-affinity nitrate uptake capability and require interaction with CrNAR2 to form functional complexes (CrNAR2/CrNRT2.1 or CrNAR2/CrNRT2.2) for HATS activity [11,12]. In *Arabidopsis thaliana*, six AtNRT2 members can interact with NRT3.1 [9]. The co-injection of the cRNA of five *NRT2* genes (*AtNRT2.2* to *AtNRT2.7*) with the cRNA of *AtNRT3.1* (*AtNAR2.1*) into *Xenopus* oocytes resulted in significant increases in uptake compared to single cRNA injections alone [9]. Among them, AtNRT2.5 forms a hetero-oligomeric complex with AtNAR2.1, exhibiting high affinity and absorption capacity [13]. In the *A. thaliana AtNRT3.1* mutation, nitrate-induced high-affinity uptake decreased by 92–96%, constitutive high-affinity uptake decreased by 34–89%, and low-affinity absorption was not affected [10]. The rice genome contains two NAR2 homologous proteins, OsNAR2.1 and OsNAR2.2, while only OsNAR2.1 can interact with OsNRT2.1 and OsNRT2.3a [14]. In rice *osnar2.1* RNAi mutants, the HATS and LATS nitrate uptake decreased by 86% and 73%, respectively [15]. Gu et al. found that oocytes injected with *Chrysanthemum morifolium CmNAR2* or *CmNRT2* alone showed no significant nitrate uptake, but co-expressed *CmNRT2* and *CmNAR2* in *A. thaliana* and oocytes significantly increased nitrate uptake rate under low N treatment [16]. Co-injected oocytes of *Hordeum vulgare HvNAR2.3* and *HvNRT2.1* have obvious ^15^N influx, while neither *HvNAR2.3* nor *HvNRT2.1* alone exhibited nitrate transport activity [17]. All of these highlight the necessity of accessory protein NRT3 in HATS.

NRT3 plays an important role in improving plant NUE. Rice lines overexpressing *OsNAR2.1* exhibited a significant increase in ^15^N uptake, biomass accumulation, total nitrogen content, and yield under both low (0.2 mM ${NO}_{3}^{-}$) and high nitrate (2.5 mM ${NO}_{3}^{-}$) conditions [18]. The heterogeneous expression of *Dianthus spiculifolius DsNRT3.1* in *A. thaliana* improved the uptake of ${NO}_{3}^{-}$ and NH_4_^+^ by promoting the expression of *AtNRT2* and eventually resulted in greater biomass compared to wild-type plants [19]. Similarly, our previous study showed that transgenic *A. thaliana* overexpressing *SoNRT3* demonstrated a markedly higher rate of ^15^N uptake and shoot fresh weight when subjected to low N treatment [20].

NRT3 proteins are also critical regulators of root development [21]. Under low nitrate concentrations, lateral root growth in the *OsNAR2.1* mutant was inhibited, while supplementing nitrate content in roots could not eliminate the inhibitory effect of the *osnar2.1* mutant on lateral roots [22]. Further studies revealed that OsNAR2.1 may affect lateral root growth by activating OsNIT1 and OsNIT2 (two proteins involved in the indole-3-acetic acid (IAA) synthesis pathway), thus changing the ratio of free-conjugated forms of IAA and affecting the polar transport and distribution of IAA [23]. A transcription factor, *OsMADS57,* a downstream gene of *OsNAR2.1*, may play a role in NRT3-mediated root development regulation [24]. 

A spinach nitrate-responsive NAR2 homologous protein (SoNRT3) was identified in our previous study [20]. Nevertheless, the molecular mechanisms underlying its function in low-N adaptation remain elusive. To elucidate the role and mechanism of SoNRT3 in enhancing plant tolerance to low-nitrate conditions, we constructed spinach and *A. thaliana* transgenic materials. By comparing the differences in phenotype, physiology, and gene expression levels between the different transgenic materials, coupled with the identification of interacting proteins, we aim to investigate: (1) the effect of SoNRT3 on nitrate uptake and its induction mechanism of the high-affinity transport system (HATS) under low-nitrate conditions; (2) the influence of SoNRT3 on root growth and to identify key genes involved in root development pathways under low-nitrate stress; (3) the relationship between SoNRT3-mediated shoot growth regulation and the nitrate reduction/assimilation pathway under low-nitrate conditions.

## 2. Results

### 2.1. Sequence, Subcellular Localization, and Evolutionary Analyses of SoNRT3

**Figure 1:**
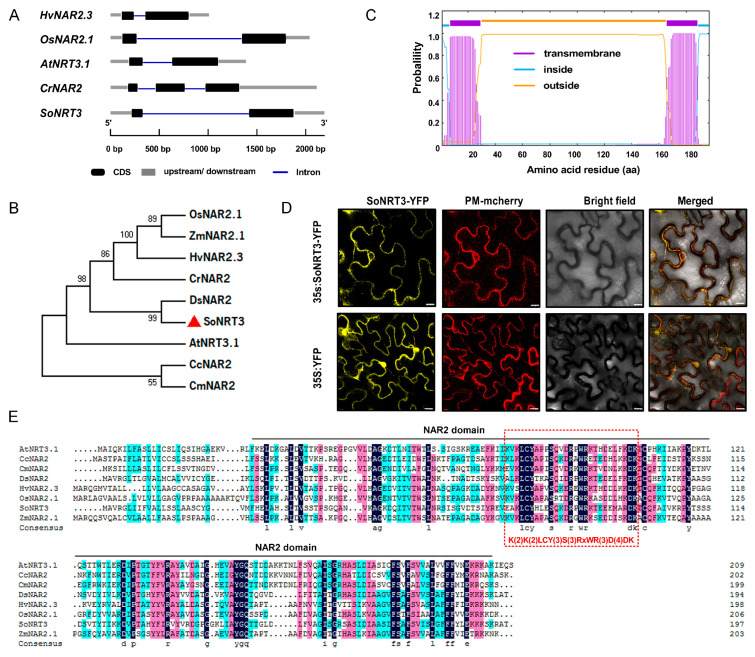
Structure analysis of spinach SoNRT3 proteins. (**A**) The structures of spinach *SoNRT3* genes. The coding DNA sequences (CDS) of a gene consist of exons (black rectangles). (**B**) Phylogeny of NAR2 polypeptides; (**C**) Transmembrane domains (TMs) of SoNRT3 were predicted by TMHMM; (**D**) Subcellular localization of the SoNRT3 protein was carried out in *N. benthamiana* leaves. Yellow fluorescent protein (YFP) was fused to the N-terminus of SoNRT3 (35S: SoNRT3-YFP). The plasma membrane (PM) was indirectly indicated by a plasma membrane marker, pm-rk CD3-1007 [25]; scale bar = 10 μm. (**E**) The alignment of SoNRT3 proteins was performed by ClustalX 2.1 (http://www.clustal.org/clustal2/) accessed on 2 March 2024. At, *A. thaliana*; Cc, *Capsicum chinense*; Cm, *Chrysanthemum morifolium*; Cr, *Chlamydomonas reinhardtii*; Ds, *Dianthus spiculifolius*; Hv, *Hordeum vulgare*; Os, *Oryza sativa*; Zm, *Zea mays*; So, *S. oleracea*.

The SoNRT3 gene comprises a single intron (Figure 1A) and encodes a 197-amino-acid protein with a predicted molecular mass of 21.68 kDa. Phylogenetic analysis indicates that SoNRT3 exhibits a higher sequence similarity to DsNAR2 (Figure 1B). Structural prediction identified transmembrane helices in SoNRT3, suggesting its possible membrane localization (Figure 1C). The transient expression of YFP-tagged *SoNRT3* in *Nicotiana benthamiana* leaves demonstrated clear plasma membrane localization, as evidenced by co-localization with the PM-mCherry marker, while free YFP showed ubiquitous distribution in the cell (Figure 1D). These results demonstrate that SoNRT3 is a plasma membrane-localized protein. MSA analysis revealed that SoNRT3 possesses a typical NAR2 family domain structure and a conserved amino acid sequence K(2)K(2)LCY(2)S(3)RxWR(3)D(4)DK, affirming its classification as a NAR2 family protein (Figure 1E).

### 2.2. SoNRT3 Improves Shoot N Utilization and Root Growth in A. thaliana

**Figure 2:**
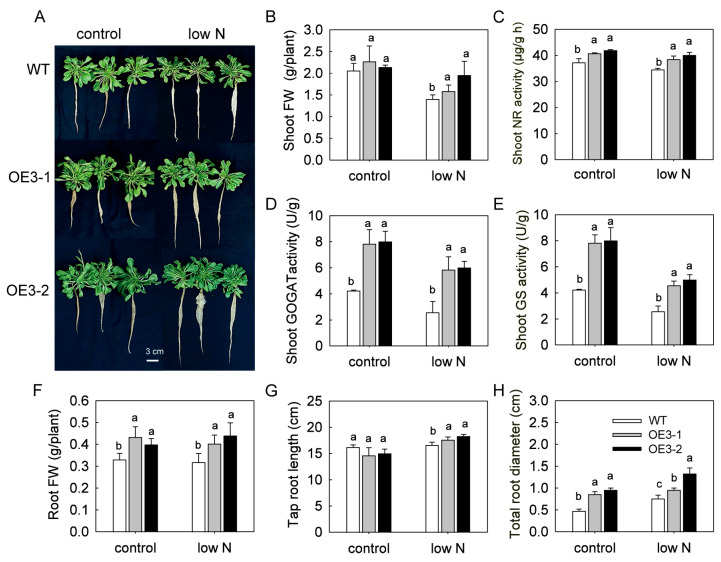
Overexpression of *SoNRT3* improves low-nitrate tolerance in *A. thaliana* under hydroponics. (**A**) Appearances of wild-type (WT) and overexpression lines (OE3) after control (5 mM ${NO}_{3}^{-}$) and low-nitrate (0.25 mM ${NO}_{3}^{-}$) treatment for two weeks, (**B**) shoot fresh weight (FW), (**C**) shoot nitrate reductase (NR) activity, (**D**) shoot glutamate synthase (GOGAT) activity, (**E**) shoot glutamine synthetase (GS) activity, (**F**) root FW, (**G**) tap root length, (**H**) total root diameter. All the values represent the means of three independent replicates ± SE (standard error). Means with different letters are significantly different in each nitrate concentration treatment (Tukey’s test, *p* < 0.05).

Wild-type (WT) and *SoNRT3*-overexpressing (OE) plants were grown hydroponically under low (0.25 mM) or high (10 mM) nitrate conditions (Figure 2A). After two weeks, OE lines showed significantly enhanced activity of key nitrogen assimilation enzymes—glutamine synthetase (GS), nitrate reductase (NR), and glutamate synthase (GOGAT)—in shoots across both nitrate treatments (Figure 2B–D). Under low nitrate supply, OE plants also exhibited greater shoot biomass accumulation (Figure 2E) and significantly longer taproots compared to WT controls. Furthermore, transgenic lines showed increased total root diameter and fresh weight under both low-nitrate and control conditions (Figure 2F–H).

### 2.3. SoNRT3 Enhanced Nitrate and Auxin-Related Gene Expression in A. thaliana Root

**Figure 3:**
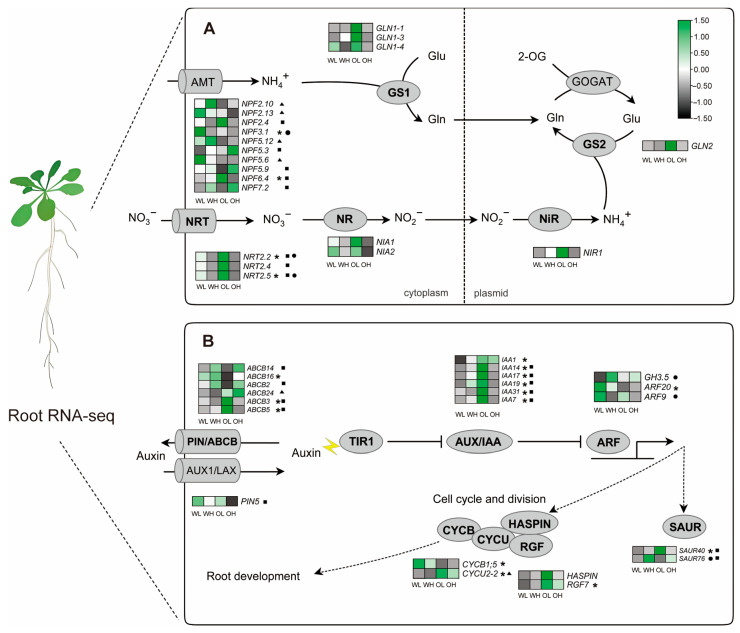
Heatmap of the expression patterns of several specific genes during the (**A**) nitrogen metabolism, (**B**) auxin signal, and cell division pathways in transgenic *A. thaliana* roots by RNA-seq. WL, low nitrate (0.25 mM)-treated wild-type plants; WH, normal nitrate (5 mM)-treated wild-type plants; OL, low nitrate (0.25 mM)-treated *SoNRT3*-overexpression (OE) plants; OH, normal nitrate (5 mM)-treated OE plants. Genes with significant differential expression (|log2(Foldchange)| >= 1, padj <= 0.05) and several specific genes (*GLN1*, *GLN2*, *NIA*, *NIR*, *and HASPIN*, |log2(Foldchange) |>= 0.80, padj <= 0.05) are presented. Green and black boxes indicate up- or down-regulation of the gene under different treatments, respectively. Asterisks, triangles, squares, and circles behind the gene names indicate great differences in gene expression between OL vs. WL, OH vs. WH, OL vs. OH, and WL vs. WH, respectively.

To investigate the effect of *SoNRT3* on the molecular changes that take place in the N metabolism and root development, we compared the transcriptome of the root system of WT and OE under control and low-nitrate treatments. RNA-seq analysis identified significant differential expression of nitrate transporters, cell cycle regulators, and auxin-related genes in the roots of OE plants compared to WT under nitrogen limitation (Figure 3, Table S1). For example, the levels of *AtNRT2.2/2.5*, *AtNPF6.4*, *AtABCB3/5*, *AtIAA7/14/17/19/31*, and *AtSAUR40* were more than two-fold higher in the roots of low-nitrate-treated OE lines (OL) compared to those of WT and normal-nitrate-treated OE (OH). Among them, the up-regulation of *AtNRT2.2* and *AtNRT2.5* was also found in low-nitrate-treated WT (WL), compared with that in normal-nitrate-treated WT (WH). The expression levels of *AtNIA1*, *AtNIA2*, *AtNIR*, *AtGLN1-1*, *AtGLN1-4*, *AtGLN2*, and *AtHASPIN* in the roots of OE were significantly higher than those in OH, though their increases were less than twofold. In contrast to WT plants, several differentially expressed genes (DEGs), including *AtNPF2.4*, *AtNRT2.4*, and *AtPIN5*, were found to be up-regulated in OL compared to OH. This observation suggests that these differential genes may be specifically involved in *SoNRT3*-mediated low-nitrate response. Under normal nitrate conditions, two genes (*AtABCB24* and *AtCYCU2-2*) showed increased expression in the roots of OE, while the expression levels of *AtNPF2.10*, *AtNPF2.13*, *AtNPF5.12*, and *AtNPF5.6* decreased when compared to those of the WT.

### 2.4. Silencing of SoNRT3 Decreases Spinach Growth in Low N Conditions

To assess the functional role of *SoNRT3* in nitrate response, virus-induced gene silencing (VIGS) was employed to suppress *SoNRT3* expression in spinach plants grown under low-nitrate (0.25 mM) conditions. Two weeks post-infiltration, the expression levels of *SoNRT3* decreased by 81% in roots and 36% in shoots, respectively (Figure S1). The silencing of *SoNRT3* significantly inhibited spinach growth under low-nitrate treatments (Table 1 and Figure S1). Compared to control plants, *SoNRT3*-silenced plants exhibited a 42% and 48% reduction in fresh weight of shoots and roots, respectively. Additionally, tap root length, lateral root number, and the ^15^${NO}_{3}^{-}$ uptake rates were also significantly reduced in the *SoNRT3*-silenced plants (Table 1). These results demonstrate that *SoNRT3* positively regulates both root and shoot development in spinach under nitrate-limiting conditions.

### 2.5. Complementation of A. thaliana Mutants

**Figure 4:**
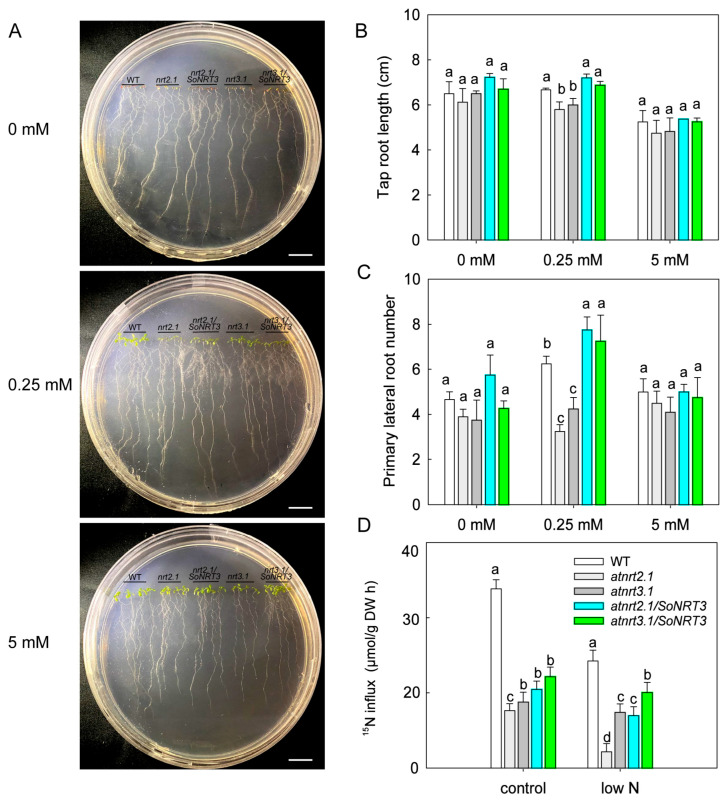
*SoNRT3* restores root growth and ^15^N uptake in low-nitrate-treated *A. thaliana* mutants *atnrt2.1* and *atnrt3.1*. (**A**) Wild type, mutants of *atnrt2.1*, *atnrt3.1*, and complemented lines grown on vertical plates with 0 mM, 0.25 mM, or 5 mM KNO_3_ for 10 days; scale bar = 1 cm. (**B**) Tap root length; (**C**) primary lateral root number; (**D**) root ^15^N influx under control (2 mM) and low N (0.25 mM) K^15^NO_3_ (99% atom). Data are means ± SE of at least three replicates. Different letters indicate significant differences within each treatment (Tukey’s test, *p* < 0.05).

To further determine the functional role of SoNRT3 in nitrate uptake and root growth, two *A. thaliana* mutant lines (*atnrt3.1* and *atnrt2.1*) and two mutant complement lines (*nrt3.1*/*SoNRT3* and *nrt2.1*/*SoNRT3*) were used (Figure 4A and Figure S2).

Under 0.25 mM nitrate treatment, mutant plants had considerably shorter tap roots than WT plants, while mutant lines overexpressing *SoNRT3* restored tap root length. The tap root length of all these lines did not differ between 0 mM and 5 mM nitrate concentration treatments (Figure 4B).

The lateral root numbers were markedly decreased in two mutant lines under 0.25 mM nitrate treatments, while two independent 35S: *SoNRT3* complementing lines (lines *nrt3.1/SoNRT3* and *nrt2.1/SoNRT3*) restored their lateral growth to or even increased it (Figure 4C).

When grown on hydroponics containing 0.25 or 2 mM nitrate for 0.5 h, a significantly lower ^15^N uptake rate was found in two mutant lines when compared with that of WT plants. The *SoNRT3* partly restored the impaired ^15^${NO}_{3}^{-}$ influx of *atnrt3.1* and *atnrt2.1* mutants under low-nitrate treatment and regained the nitrate uptake rates by an average of 1.2 and 1.4 times, respectively. However, the ^15^N influx was only partly restored by *SoNRT3* in the *atnrt2.1* mutants under 2 mM nitrate treatments, with 41% less than those of the WT, respectively (Figure 4D).

### 2.6. SoNRT3 Interacts with SoNRT2a

**Figure 5:**
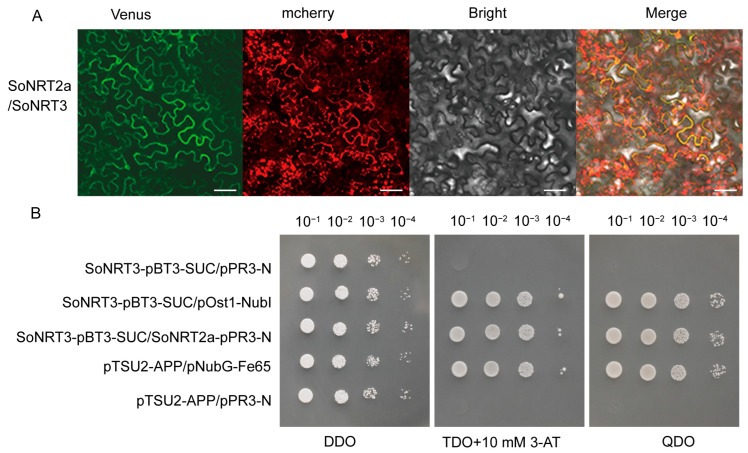
Analysis of the interaction and expression patterns of SoNRT3 and SoNRT2a. (**A**) BiFC assay detected the interactions of SoNRT3 (fused with the C-terminal fragment of Venus) and SoNRT2a (fused with the N-terminal fragment of Venus) in transiently transformed *N. benthamiana* leaves. Scale bar = 20 μm. (**B**) Yeast two-hybrid analyses of the interaction between SoNRT3 and SoNRT2a. Serial dilutions of yeast transformant cells harboring the indicated plasmids were spotted on double dropout medium (DDO, SD/–Leu/–Trp), triple dropout medium (TDO, SD/-His/-Leu/-Trp) containing 10 mM 3-AT, and quadruple dropout medium (QDO, SD/–Ade/–His/–Leu/–Trp) at 30 °C for 4 days, respectively. SoNRT3-pBT3-SUC/pPR3-N (self-activation); SoNRT3-pBT3-SUC/pOst1-NubI (functional verification); SoNRT3-pBT3-SUC/SoNRT2a-pPR3-N (experimental group); pTSU2-APP/pNubG-Fe65 (positive control); pTSU2-APP/pPR3-N (negative control).

Several SoNRT2 members that may interact with SoNRT3 were predicted by STRING (Figure S3A). Among them, a spinach NRT2 member, SoNRT2a, initially named SoNRT2.4, was selected for further study. Phylogenetic analysis indicates that SoNRT2a exhibits the highest sequence similarity to AtNRT2.5 (Figure S3B). Bimolecular fluorescence complementation (BiFC) analysis showed that the *N. benthamiana* leaf cells expressing *SoNRT2a-nVenus* and *SoNRT3-cVenus* exhibited strong YFP complementation (Figure 5A). In contrast, cells transformed with either *SoNRT2a-nVenus/cVenus*, *nVenus/SoNRT3-cVenus*, or *nVenus/cVenus* emitted no fluorescence. This indicated that SoNRT3 can interact with SoNRT2a in vivo.

Yeast two-hybrid heterologous expression was also used to assess possible interaction between SoNRT3 and SoNRT2a. Yeast was transformed with SoNRT3 as bait and SoNRT2a as prey. As shown in Figure 5B, high transformation efficiency was confirmed on double dropout medium (DDO, SD/–Leu/–Trp). Only positive controls and bait–prey co-transformants grew on selective media (TDO with 10 mM 3-AT and QDO), whereas the negative control did not survive. This result showed that SoNRT3 interacted with SoNRT2a, which was consistent with the results obtained by BIFC.

### 2.7. Nitrate Response Expressions of SoNRT3 and SoNRT2a in Spinach

**Figure 6:**
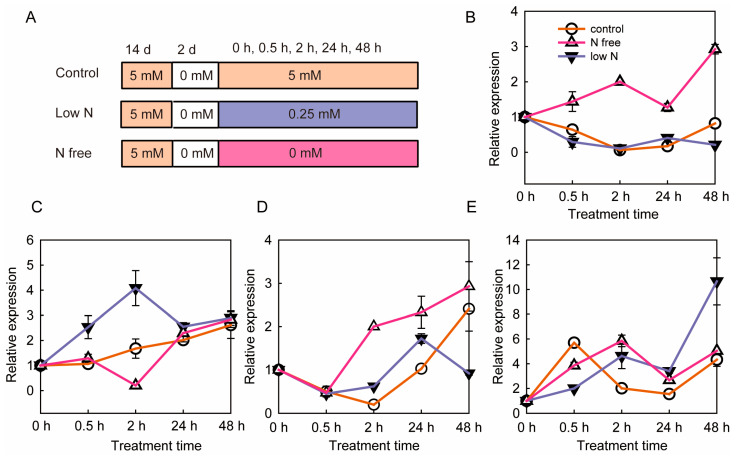
Expression patterns of *SoNRT3* and *SoNRT2a* in response to different nitrate concentrations. (**A**) Design scheme of nitrate treatment for gene expression analysis. Control, 5 mM ${NO}_{3}^{-}$; low N, 0.25 mM ${NO}_{3}^{-}$; N free, 0 mM N; nitrate response expression patterns of spinach SoNRT3 expression in (**B**) shoot and (**C**) root, respectively. Nitrate response expression patterns of spinach *SoNRT2a* expression in (**D**) shoot and (**E**) root, respectively. Relative expression was calculated using the expression at 0 h of the control group as the control. All values represent the means of three independent replicates ± SE.

The nitrate response expressions of *SoNRT3* and *SoNRT2a* in spinach were examined by using qRT-PCR (Figure 6B–F). Both *SoNRT3* and *SoNRT2a* can be expressed in both shoot and root and were sensitive to nitrate supply. Under low-nitrate treatments, shoot *SoNRT3* and *SoNRT2a* transcripts accumulated progressively, reaching maximal levels at 48 h. In roots, *SoNRT3* expression was rapidly induced at 2 h, whereas *SoNRT2a* showed significant induction at both 2 h and 48 h of low nitrogen treatment.

## 3. Discussion

The functional role of SoNRT3 in shoot biomass and nitrate uptake was identified in a previous study. However, the mechanism by which SoNRT3 regulates low N response is not fully understood. In this study, transgenic overexpression/silencing, mutation compensation, transcriptomics, and protein interactions were used to elucidate the molecular mechanism of SoNRT3 in spinach response to low nitrogen stress.

As is known, NRT3 proteins cannot uptake nitrate, as they are not transporters but are partner proteins of transporters from the NRT2 family [9,16,17]; therefore, we propose that the increased nitrate uptake by SoNRT3 may be attributed to the interaction of SoNRT3 with multiple NRT2s in *A. thaliana* [10,26]. That is, SoNRT3 possesses an AtNRT3.1-like function in HATS. In this study, we observed that the *atnrt3.1* mutant partially compensates for ^15^N nitrate uptake by supplementing SoNRT3. Moreover, the expression of *AtNRT2.2* and *AtNRT2.5* in the low-nitrate-treated *SoNRT3*-overexpressing plants was greatly increased, as revealed by transcriptome analysis. It is well known that AtNRT2.2 and AtNRT2.5 are the two major contributors to high-affinity ${NO}_{3}^{-}$ influx in *A. thaliana*, accounting for approximately 19% to iHATS and 63% to cHATS, respectively [13,26]. Therefore, the increased nitrate uptake may result from the up-regulation of these high-affinity nitrate transporters. Subsequent mutant complementation experiments conducted on the *atnrt2.1* mutant further supported this hypothesis. The reduced ^15^N uptake by the *NRT2.1* mutation may be compensated by *SoNRT3*-induced *AtNRT2.2*. Previous studies suggested that AtNRT2.2 can provide partial compensation of iHATS when *AtNRT2.1* is silenced [26]. Furthermore, silencing *SoNRT3* resulted in a greatly reduced ^15^N uptake in spinach. All of this suggests that SoNRT3 may not only function as a chaperone of high-affinity transporters but is also capable of inducing HATS independently.

Besides nitrate uptake, enzyme activities related to nitrogen reduction and assimilation were greatly up-regulated in the overexpressed lines. This indicates that *SoNRT3* overexpression not only promotes nitrate uptake but also enhances N assimilation capacity, which allows more nitrogen to be utilized for shoot and root biomass accumulation and ultimately improves NUE. To further elucidate the role of SoNRT3 in promoting plant growth, a VIGS system was developed to silence *SoNRT3* in spinach. The inhibition of the *SoNRT3* led to a significant reduction in nitrate uptake and shoot and root biomass of spinach seedlings, confirming that *SoNRT3* is a promising candidate gene for enhancing NUE in spinach under conditions of nitrogen limitation.

The role of SoNRT3 in root development needs further emphasis. Unlike *DsNRT3.1*, which only promoted root growth under N-deficient conditions [19], the overexpression of *SoNRT3* markedly promotes root (especially lateral root) growth, regardless of whether nitrate supply is low or normal. Furthermore, silencing *SoNRT3* resulted in reduced root biomass and a significant decrease in the number of lateral roots in spinach, thereby confirming the essential function of SoNRT3 in lateral root development. It is understood that root development involves auxin biosynthesis, nitrate signaling, and cell differentiation [27,28,29]. Transcriptome analysis actually found the up-regulation of genes involved in auxin signal transduction, cell division, and root growth in transgenic *SoNRT3* lines. For example, the levels of *IAA17* and *SAUR40* in the roots of low-nitrate-treated OE plants were higher than those of WL. Among them, the IAA17 participates in auxin response, cell elongation, and root development in *A. thaliana* [30]. *SAUR40* is a member of small auxin up-RNAs (SAURs) family, which was originally identified as a group of auxin-responsive genes. Current studies suggest that SAURs play key roles in integrating multiple signals into distinct growth and developmental responses, i.e., light and auxin-mediated cell elongation and growth [31]. These findings suggest that SoNRT3 promotes lateral root development mainly by affecting auxin response pathways.

The potential interaction between SoNRT3 and SoNRT2 was investigated. Initially, we examined the sequence of SoNRT3 and identified a conserved motif (K(2)K(2)LCY(2)S(3)RxWR(3)D(4)DK) characteristic of the NRT3 family [17]. Within this motif, two amino acids (D and R) are critical for the interaction between NRT2 and NRT3. The replacement of D and/or R has been shown to impair this interaction and inhibit the HATS activity [32,33]. In spinach, these two essential amino acids are conserved in the SoNRT3 sequence, indicating that the SoNRT3 may interact with the protein from the NRT2 family.

BiFC and membrane yeast two-hybrid assays confirmed the interaction between SoNRT3 and SoNRT2a, with their interaction localized specifically to the plasma membrane. This finding was consistent with previous reports on the plasma membrane localization of NRT2/NAR2 complexes in other plant species, including *A. thaliana* [26], *H. vulgare* [34], and *C. morifolium* [16]. Interestingly, unlike SoNRT3 localized in the plasma membrane, OsNAR2.1 is found in both the membrane and the cytoplasm [33], whereas CmNAR2 is found throughout the cell [16]. However, when co-expressed with their respective NRT2 proteins, both OsNAR2.1 and CmNAR2 localized to the plasma membranes. This suggests that, regardless of where NRT3 is located, the plasma membrane is most likely the functional location for these components in HATS. It should be noted that the co-expression of wheat *TaNRT2.5-3B* and *TaNAR2.1-6B*, which are implicated in the regulation of seed nitrate accumulation and seed viability, was localized in the tonoplast rather than in the plasma membrane [35]. This finding suggests that the interaction between these two components is closely related to their specific functions.

*SoNRT2a* had a similar expression profile to *SoNRT3* in response to N supply, both being induced by low nitrogen in roots and long-term N deficiency in shoots. A similar N-responsive expression pattern between NRT2 and NRT3 was also found in *A. thaliana* [9,10], *O. sativa* [15], *C. morifolium* [16], *Medicago truncatula* [36], and *C. chinense* [37]. For example, the transcript levels of both *AtNRT3.1* and *AtNRT2.1* were induced by low N (0.25 mM) and short-term N deficiency and inhibited by high N (2.5 mM) [9,10]. The expression of *OsNAR2.1* and *OsNRT2.1/2.2/2.3a* was induced by low (0.2 mM, 2.5 mM) and high (5 mM) nitrate and inhibited by high ammonium (2.5 mM) [15]. The similar temporal profiles suggested that SoNRT3 and SoNRT2a may act in concert during nitrate uptake.

## 4. Materials and Methods

### 4.1. Plant Materials and Treatments

Spinach sibling inbred line SP75, *A. thaliana* (ecotype Columbia, wild type, and transgenic lines), and *A. thaliana* mutants of *atnrt3.1* (SALK_043672C) and *atnrt2.1* (SALK_035429C) were used in this study. All plants were grown in an environmental chamber with light/dark periods of 8 (25 °C)/16 h (20 °C), relative humidity of 60–70%, and a light intensity of 200 µmol/m^2^ ·s. When Spinach and *A. thaliana* reached the stage of 2–4 true leaves, plants of uniform size were selected for the nitrate treatments under hydroponics. The nutrient solution for plant growth contained 5 mM KNO_3_, 1 mM KH_2_PO_4_, 0.5 mM MgSO_4_, 0.50 mM CaCl_2_, 0.05 mM NaFeEDTA, 0.02 μM (NH_4_)_6_Mo_7_O_24_, 46 μM H_3_BO_3_, 0.31 μM CuSO_4_, 0.77 μM ZnSO_4_, and 9.15 μM MnCl_2_. The pH of the nutritional solutions was adjusted to 5.8 ± 0.2. The K_2_SO_4_ was used in place of KNO_3_ to compensate for the K^+^ deficit in free (0 mM N) or low-nitrate (0.25 mM ${NO}_{3}^{-}$) treatment. The rest of the nutritional solution remained unchanged. When *A. thaliana* mutants’ seedlings were grown on agar plates, the nutrients were the same as those under hydroponics except for the addition of 2.5 mM MES, 1 g L^−1^ sucrose, and 1 g L^−1^ agar.

### 4.2. Isolation and Analysis of SoNRT3

The coding sequence (CDS) of *SoNRT3* (SOV1g041920.1) was amplified from SP75 seedling cDNA. Primer sequences are listed in Table S2. Homologous protein sequences were retrieved from NCBI (https://www.ncbi.nlm.nih.gov, accessed on 20 January 2024). Multiple sequence alignment and phylogenetic analysis were conducted using DNAMAN and MEGA software, respectively. Gene structure was analyzed using GSDS 2.0, while transmembrane domains were predicted with TMHMM 2.0.

### 4.3. Subcellular Localization of SoNRT3

The *SoNRT3* cDNA was cloned into the pCAMBIA1300 vector containing a YFP reporter, with pm-rk CD3-1007-mCherry serving as a plasma membrane marker [25]. The 35S: SoNRT3-YFP fusion construct was transformed into *Agrobacterium tumefaciens* GV3101 and transiently expressed in *N. benthamiana* leaves. The infiltration buffer contained 10 mM MES (pH 5.6), 10 mM MgCl_2_, and 100 μM acetosyringone. Confocal imaging (Leica TCS SP8) was performed at 72 h post-infiltration to visualize YFP fluorescence.

### 4.4. Bimolecular Fluorescence Complementation (BiFC) Assay

The BiFC assay was conducted as previously described [38]. Initially, the CDSs of *SoNRT2a* (SOV1g045270.1) and *SoNRT3* were cloned into pYL322d1/nVenus and pUC19/cVenus, respectively. Subsequently, the two fluorescent fusion protein expression cassettes were assembled into a single plasmid to create pYL1300/SoNRT2a-nVenus-SoNRT3-cVenus. Following this, the co-expression vectors were introduced into *A. tumefaciens* GV3101 (pSoup-p19) and co-infiltrated into the leaves of *N. benthamiana*. After 48 h of incubation, confocal laser microscopy (Olympus FV3000, Tokyo, Japan) was employed to monitor Venus’s expression.

### 4.5. Split-Ubiquitin Membrane Yeast Two-Hybrid System

The membrane yeast two-hybrid (Y2H) assay was also performed to investigate the potential interaction between SoNRT3 and SoNRT2a. The CDS of *SoNRT3* (remove the N-terminal signal sequence) and *SoNRT2a* were cloned into the pBT3-SUC (bait vector) and pPR3-N (prey vector), respectively. Bait construct expression was validated through co-expression with the pOST1-NubI control plasmid. Preliminary tests with the empty prey vector pPR3-N demonstrated that 10 mM 3-AT effectively suppressed basal HIS3 reporter activity in minimal media. For interaction assays, SoNRT3-pBT3-SUC and SoNRT2a-pPR3-N were co-transformed into yeast strain NMY51 and selected on TDO (SD/-His/-Leu/-Trp) + 10 mM 3-AT and QDO (SD/-Ade/-His/-Leu/-Trp). The pTSU2-APP/pNubG-Fe65 pair served as positive control, while SoNRT3-pBT3-SUC/pPR3-N and pTSU2-APP/pPR3-N combinations provided negative controls.

### 4.6. Overexpression of SoNRT3 in A. thaliana

The CDSs of *SoNRT3* were cloned into the pEarleyGate100 vector to generate 35S promoter-driven overexpression constructs. *A. thaliana* transformation was performed via *A. tumefaciens*-mediated floral dip using GV3101 strains harboring these constructs [20]. The putative transformants were selected on kanamycin-supplemented MS medium (100 mg/L), with transgene integration verified by PCR. The T3-positive transformants were used for subsequent analyses.

### 4.7. Virus-Induced Gene Silencing (VIGS)

A 591 bp fragment specific to *SoNRT3,* with no mismatches identified in other SoNRTs, was selected for VIGS. The primers utilized are detailed in Table S2. The PCR-amplified fragment was subsequently cloned into a pTRV2 vector using a PstI restriction site using a seamless assembly kit (Uni-Seamless Cloning and Assembly Kit, TransGen Biotech, Beijing, China). The VIGS assay was performed as previously reported with a minor modification [39]. The *A. tumefaciens* strain GV3101 (pSoup-p19), harboring either pTRV1, pTRV2, or pTRV2: SoNRT3, was adjusted to an OD600 of 0.6 and mixed at a ratio of 2:1 before infiltrating hydroponically grown spinach leaves at the two-leaf stage. Phenotypic observations were conducted after two weeks.

### 4.8. Gene Expression Analysis

Total RNA was isolated from plant tissues using TRIzol reagent (Invitrogen, Carlsbad, CA, USA), followed by cDNA synthesis with PrimeScript™ RT reagent Kit (Takara, Shiga, Japan). Quantitative real-time PCR (qRT-PCR) was performed with three biological replicates as previously described [20], with spinach 18S rRNA and *A. thaliana* Actin serving as reference genes. Gene expression levels were normalized and quantified using the 2^−ΔΔCT^ method [40]. Gene-specific primers for qRT-PCR are listed in Table S2.

For RNA-seq, the RNA from WT and OE lines treated with different nitrate concentration treatments was extracted and used to construct the sequencing libraries as described previously [20]. The clean reads were aligned to the *A. thaliana* reference genome. Differentially expressed genes (DEGs) were identified using DESeq2 with thresholds of |log2FC| ≥ 1 and adjusted *p*-value < 0.05. The corresponding sequencing data were summarized in Table S3.

### 4.9. Root ^15^N-Nitrate Influx, Uptake Rate, and Accumulation

The ^15^N influx of transgenic, non-transgenic lines, and mutants was assayed as described previously [2,41]. The plants grown in hydroponics were initially transferred to a solution of 0.1 mM CaSO_4_ for 1 min, followed by immersion in a hydroponic solution containing either 0.25 or 2 mM ^15^${NO}_{3}^{-}$ (K^15^NO_3_, 99% of atom, Cambridge Isotope Laboratories, Tewksbury, MA, USA) for 0.5 h. After washing the seedlings in 0.1 mM CaSO_4_ for 1 min, they were separated, freeze-dried, and analyzed for ^15^N excess using a VarioEL III IRMS (Elementar Analysensysteme GmbH, Hanau, Germany), following the methodology outlined previously [2,41]. The net ^15^N influx of shoots or roots was calculated from the ^15^N excess of the shoots/roots per hour.

### 4.10. Physiological Measurements and Observations

At harvest, root images were analyzed in WinRHIZO (Regent Instruments, Quebe, QC, Canada) to acquire the total root length, tap root length, and primary lateral root number. After photographing, shoots were separated from the roots, and the total root diameter, shoot fresh weight, and root fresh weight were recorded manually. The maximal extent of the root system in a horizontal direction was defined as the total root diameter.

The soluble protein contents and the enzymatic activities of nitrate reductase (NR), glutamine synthetase (GS), and glutamate synthase (GOGAT) were quantified using detection kits from Sangon Biotech, Shanghai, China, following the manufacturer’s protocols.

### 4.11. Statistical Analysis of Data

Statistical analyses were performed using IBM SPSS 29.0 (SPSS, Armonk, NY, USA). Treatment differences were assessed by one-way ANOVA followed by Tukey’s test (*p* < 0.05). Data are presented as means ± standard error (SE).

## 5. Conclusions

In summary, this study demonstrates that SoNRT3 enhances plant nitrogen uptake, lateral root development, and shoot growth in response to low-nitrate conditions, highlighting the crucial role of SoNRT3 in low-nitrate tolerance. Notably, unlike NRT3 homologs in other species, which solely function as chaperones, SoNRT3 not only interacts with SoNRT2a as a chaperone but also independently activates the HATS to facilitate nitrogen uptake. The development of roots, especially lateral roots, further enhanced N uptake under low N. Transcriptome analysis further suggested that SoNRT3 may modulate lateral root growth by influencing auxin signaling and cell differentiation. The improved nitrogen use efficiency observed in SoNRT3-overexpressing plants may be attributed to increased activities of nitrate reduction and assimilation enzymes, as well as the up-regulation of their corresponding genes. Given the current limitations in spinach stable genetic transformation, *A. thaliana* was used as the primary model system in this study. Thus, certain findings warrant further validation in spinach. Despite this, our results strongly suggest that SoNRT3 could serve as a promising target for future spinach breeding programs aimed at enhancing nitrogen use efficiency while minimizing nitrate accumulation.

## Figures and Tables

**Table 1:** Comparison of control plants (TRV2) and *SoNRT3*-silenced spinach (TRV2-SoNRT3) under 0.25 mM nitrate treatment. Data are means ± SE of at least three replicates.

	TRV2	TRV2-SoNRT3
Shoot FW (g)	0.91 ± 0.21	0.53 ± 0.04
Root FW (g)	0.23 ± 0.08	0.12 ± 0.03
Tap root length (cm)	25.24 ± 0.45	13.80 ± 1.30
Lateral root number	73.67 ± 6.11	37.67 ± 5.13
^15^${NO}_{3}^{-}$ uptake rate (μmol/gDW h)	22.32 ± 2.34	18.01 ± 1.75

## Data Availability

The data supporting this study’s findings are available in this article’s Supplementary Materials.

## Supplementary Materials

The following supporting information can be downloaded at: https://www.mdpi.com/article/10.3390/plants14142126/s1, Figure S1: The relative expression of *SoNRT3* and appearances of control plants (TRV2) and *SoNRT3*-silenced spinach (TRV2-SoNRT3) under 0.25 mM nitrate treatment; Figure S2: Complementation of *A. thaliana* mutants by *SoNRT3*. (a) Mutant verification; (b) PCR amplification of transgene in wild-type (Columbia-0), atnrt3.1, arnrt2.1, and complement lines (*arnrt2.1*/*SoNRT3* and *arnrt3.1*/*SoNRT3*); Figure S3: Prediction of SoNRT3-interacting proteins by SRTING (a) and neighbor-joining phylogenetic trees of NRT2 from spinach and *A. thaliana* (b); Table S1: The differently expressed genes in different nitrate treatment of transgenic *A. thaliana*; Table S2: Primers used in this study; Table S3: (a) Pearson’s correlation between biological replicates of RNA-seq; (b) summary of mapping reads of the RNA-Seq; (c) number of DEGs compared between different treatments.

## Abbreviations

The following abbreviations are used in this manuscript:

N	Nitrogen
NRT	Nitrate transporter
NUE	Nitrogen use efficiency
BIFC	Bimolecular fluorescence complementation
