# Supplementary material for: Spinach SoNRT3 Interacts with SoNRT2a to Improve Low-Nitrogen Tolerance via Nitrate Uptake and Root Growth
Source: Plants (Basel). 2025 Jul 10;14(14):2126. doi: 10.3390/plants14142126 (PMC12299417; doi:10.3390/plants14142126)
Supplement: Supplementary file 1 [file plants-14-02126-s001.zip › Supplementary Figures.pdf]

Spinach SoNRT3 interacts with SoNRT2a to improve low-nitrogen tolerance via nitrate uptake and root growth

Supplementary Figures

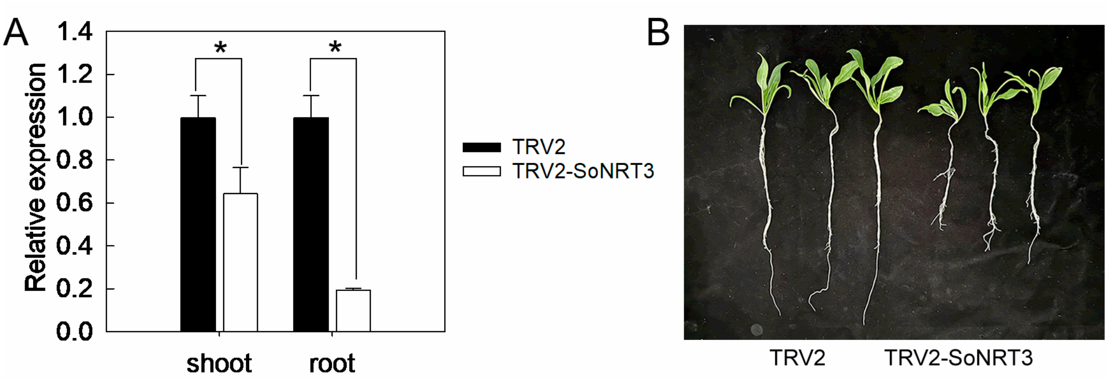

Figure S1 The relative expression of *SoNRT3* and appearances of control plants (TRV2) and *SoNRT3*-silenced spinach (TRV2-SoNRT3) under 0.25 mM nitrate treatment.

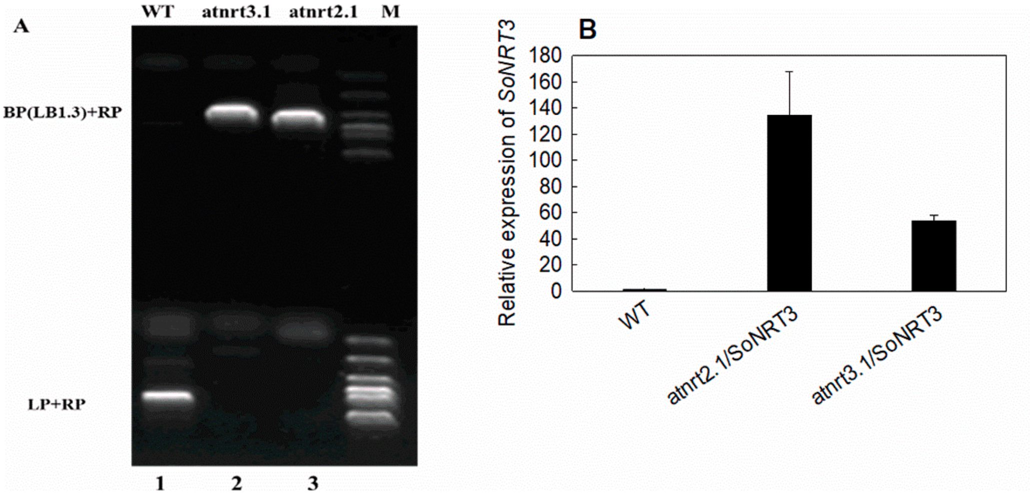

Figure S2: Complementation of Arabidopsis mutants by SoNRT3. (a) Mutant verification; (b) PCR amplification of transgene in wild type (Columbia-0), *atnrt3.1*, *arnrt2.1,* and complement lines(*arnrt2.1/SoNRT3* and *arnrt3.1/SoNRT3*).

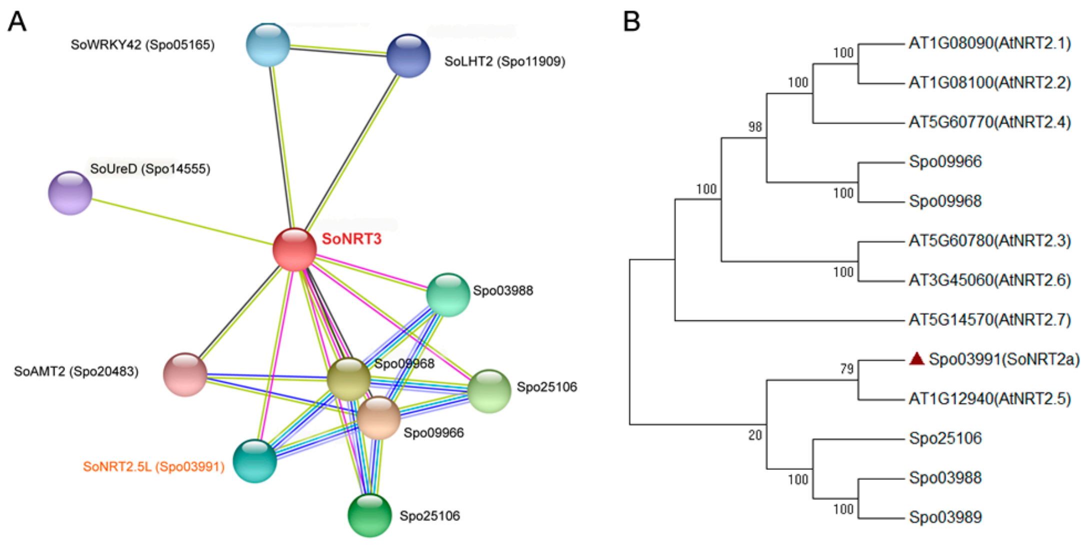

Figure S3 Prediction of SoNRT3 interacting proteins by SRTING (a) and Neighbor-joining phylogenetic trees of NRT2 from spinach and *Arabidopsis* (b).
